# Spinal Manipulative Therapy on a Healthy Population: Protocol for a Randomized Blinding Feasibility Trial

**DOI:** 10.2196/85956

**Published:** 2026-03-27

**Authors:** Margaret Sliwka, Tyson Perez, Ahmed Qazi, Stephanie Sullivan

**Affiliations:** 1 Dr. Sid E. Williams Center for Chiropractic Research, Life University, 1429 Lucile Ave, Building 900, Ste 910, Marietta, GA, 30067, United States, 1 678-331-4471

**Keywords:** double-blind, chiropractic, manual therapy, randomized controlled trial, gait analyses

## Abstract

**Background:**

Few manual chiropractic high velocity, low amplitude (HVLA) type shams have been validated in research. The proposed project is a randomized controlled trial (RCT) designed to assess a novel, full-spine, manual sham chiropractic maneuver and its blinding success.

**Objective:**

This study aims to evaluate the blinding integrity of participants receiving a genuine or sham chiropractic maneuver. We will also be evaluating the effects of genuine chiropractic treatments relative to sham chiropractic treatments by measuring several neurophysiological mechanisms.

**Methods:**

Participants (N=60) will be recruited from in and around Marietta, Georgia, United States. They will undergo a chiropractic physical exam and health history review with a licensed chiropractor and be randomized to either a sham or genuine chiropractic group (1:1 ratio). Participants, outcome assessors, and data analysts will be blinded to group allocation. The genuine group will receive diversified HVLA chiropractic spinal manipulative therapy (SMT), while the sham group will receive a novel chiropractic HVLA-emulating therapy. Each participant will attend 2 sessions spaced 1 week apart. Assessments will consist of blinding surveys postsessions (both visits) and presession (second visit). Additionally, we will measure gait parameters. The primary outcome of interest is blinding measured via the Bang Blinding Index (Bang BI). Secondary aims include measuring blinding via the James Blinding Index (James BI) and exploring the potential differential effects of genuine versus sham SMT on gait parameters.

**Results:**

This study is being supported and internally funded by the Dr. Sid E. Williams Center for Chiropractic Research at Life University in Marietta, Georgia, United States. Our study was prospectively registered on clinicaltrials.gov (NCT06931600) on March 28, 2025, and the first participant was enrolled on May 5, 2025. As of December 10, 2025, 18 participants have completed the trial. Projections for completing data collection, data analysis, and manuscript submission are Summer 2026, Fall 2026, and Winter 2027, respectively.

**Conclusions:**

The significance of the current RCT will be in its ability to inform whether our novel, full-spine, manual sham SMT protocol successfully blinds participants, suggesting feasibility for future clinical trials, as well as assessing for secondary outcome measures between groups.

## Introduction

Randomized controlled trials (RCTs) are widely regarded as essential in evidence-based health care. To accurately evaluate treatment effects, RCTs often rely on sham control interventions. These controls are designed to mimic the nonspecific aspects of treatment, such as provider attention, touch, and therapeutic interaction, without delivering the active therapeutic component [1]. By using shams, researchers are better able to distinguish between the specific effects of an intervention versus those changes resulting from other factors relating to psychological response or contextual markers. When it comes to spinal manipulative therapies (SMTs), the treatment itself cannot be easily masked, and sham interventions can therefore play a crucial role in minimizing bias.

Developing credible sham interventions for SMTs is a challenging endeavor. SMTs and other manual therapies usually involve direct physical contact, as well as distinct movements or methodologies, making it difficult to design a control that is both convincing to participants and yet lacking any kind of specific therapeutic effect. Therapist touch, timing, and intention are hard to simulate without unintentionally introducing some active input [2,3]. Additionally, the therapeutic environment, which involves verbal cues, confidence of the provider, and ritual, has previously been shown to produce clinical outcomes that are independent of the actual physical intervention [4,5]. These complexities make it difficult to create a standardized sham SMT protocol and can distort the interpretation of treatment effectiveness.

In chiropractic research, several sham SMT procedures have been tested. These range from superficial contact without thrust to zeroed-out mechanical devices or maneuvers mimicking SMT (also known as a chiropractic “adjustment”) but applied in nontherapeutic directions [6,7]. Despite these efforts, systematic reviews have found significant variation in how convincing and inert these controls are [8-10]. Many trials also fail to report on or verify whether participants were truly blinded and unaware of their intervention assignment, undermining the strength of the study findings [11,12]. Consequently, there remains a need for standardized, validated sham SMT interventions that are both perceptually credible and limiting in physiological input for the participants.

Two widely referenced tools in manual therapy studies are the Bang Blinding Index (Bang BI) and the James Blinding Index (James BI), which offer distinct approaches to accurately measuring blinding [13,14]. The Bang BI examines blinding separately for each treatment group and evaluates both the degree and direction of potential bias. In contrast, the James BI provides an overall interpretation of blinding by comparing correct guesses to what would be expected by chance, where “don’t know” responses are regarded as optimal [13,14]. As such, the Bang BI helps with the identification of group-specific bias patterns, whereas the James BI provides a more overall view of blinding success [13-15]. When used together, these 2 indices can strengthen confidence in a study’s internal validity by offering complementary interpretations of the results. Recent studies have evaluated the use of these 2 indices and recognized the feasibility, utility, and importance of them in manual therapy trials [15,16]. These studies also highlighted the importance of testing blinding in manual therapy research, especially in chiropractic trials where tactile and treatment context cues may influence participants’ expectations. Blinding efficacy is fundamental to maintaining the validity of clinical trials, especially when comparing the differences between sham and genuine SMTs.

In addition to blinding efficacy, we are also interested in exploring the possible neurophysiological mechanisms potentially responsive to change following chiropractic care. It is therefore relevant to quantify and assess movement-related measures such as gait parameters to elucidate the definitive effects of such therapeutic treatments. Contemporary evidence involving genuine SMTs has provided evidence to suggest such therapies result in improvements in several gait parameters, including step length and gait symmetry [17]. However, there is limited data comparing gait outcomes between genuine SMTs and shams. Therefore, we aim to expand the literature on the potential impacts of SMT on movement-related outcomes within the context of a putatively robust study design.

In this RCT, we aim to evaluate several things. Our primary outcome measure will consist of evaluating the blinding integrity of participants (as measured via the Bang BI). Our secondary outcome measures will be to evaluate the blinding integrity of participants (as measured by James BI), as well as to investigate the potential effects of genuine SMT relative to sham SMT via gait kinematics and biomechanics.

## Methods

### Trial Design

This study is a randomized, blinded (participants, outcome assessors, and data analysts), sham-controlled, parallel-group (2-arm) trial with an allocation ratio of 1:1.

### Study Population and Setting

Our target population is healthy adults living in and around Marietta, Georgia, United States. This study will take place at Life University’s Dr. Sid E Williams Center for Chiropractic Research (CCR) located in Marietta.

### Eligibility Criteria

The detailed eligibility criteria are presented in Textbox 1.

Textbox 1. **Inclusion and exclusion requirements for participant selection in this study.**

Eligibility criteria were defined to specify the inclusion and exclusion requirements for participant selection in this study:

- 18‐60 years of age
- No history of stroke or transient ischemic attack or the symptoms, including:

- Dizziness or vertigo
- Tinnitus (ringing in the ears)
- Visual, sensory, or motor disturbances

- No new pattern headache complaint
- No recent whiplash injury (within 3 months)
- No spinal fractures or dislocations
- No disc problems with radiating symptoms to the arms or legs
- No severe degenerative joint disease in the spine
- No connective tissue disorders
- No primary fibromyalgia
- No metabolic or metaplastic bone disease
- No history of cervical, thoracic, or lumbar spine surgery
- No uncontrolled high blood pressure or vascular disease
- No current use of anticoagulant therapy
- No diagnosed condition that causes fainting during postural changes, such as Postural Orthostatic Tachycardia Syndrome (POTS) or orthostatic hypotension
- No current use of short-acting benzodiazepines, including midazolam and triazolam
- No change in medications in the past 6 weeks or intentions to change medications during the study
- Inability to walk unassisted on a treadmill
- No pacemakers or known heart conditions that influence the electrical or mechanical function of the heart, such as severe heart valve disease
- Not a doctor of chiropractic or a doctor of chiropractic student enrolled in the 4th quarter or above
- No present or self-reported pregnancy
- No chiropractic care in the 2 weeks prior to participation
- Did not participate in our prior feasibility study (Perceptions and experiences following a single session of simulated or genuine high velocity, low amplitude [HVLA] manual chiropractic adjustments)

### Explanation for the Choice of Comparators

We have developed a manual, full-spine, sham SMT for use in future RCTs and are endeavoring to test its credibility. A credible sham control will allow SMT researchers to elucidate any potential specific effects and help address concerns of generally weak methodological designs in SMT-related trials [18,19].

### Recruitment

Participants will be recruited via targeted advertising (eg, social media advertisements, emails, and flyers) with an invitation to participate in a Life University trial involving SMT. Recruitment materials will briefly describe the study and direct potential participants to a short online screening form via a link or QR code. Individuals who complete the online screen and meet the eligibility requirements will be contacted via email and/or phone and asked to attend an in-person session at Life University’s Dr. Sid E Williams CCR. Recruitment will continue until our target sample size is met and is expected to take up to 12 months. To help foster recruitment efforts, participants will receive up to US $75 in gift cards total following their participation as reimbursement for their time.

### Participant Timeline

**Figure 1.**
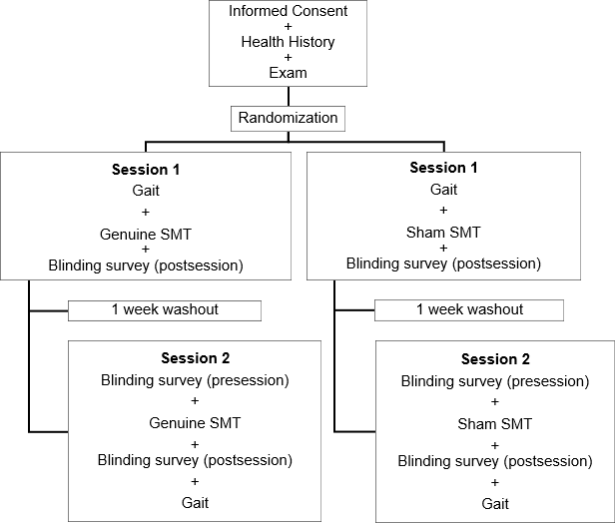
Study flow chart diagram. SMT: Spinal Manipulative Therapy.

This trial will require 2 sessions, one week apart. The participants will receive the same intervention (ie, genuine or sham SMT) at both sessions. The study flow is outlined in Figure 1.

### Demographic Information, Health History, and Anthropometric Measurements

Participants will be given a quiet room and asked to provide their demographic information and health history on an electronic tablet. Participants will then have their anthropometric measurements (ie, height and weight) recorded.

### Chiropractic Exam

During their initial session, participants will be escorted to an exam room and introduced to their attending chiropractor (MS or AJ). MS or AJ will discuss the participant’s health history. Orthopedic and/or neurological assessments may be performed to rule out red flags. If during the physical exam MS or AJ feels X-rays are indicated, finds contraindications to chiropractic care (ie, red flags), or fails to locate any spinal segmental dysfunctions, the candidate will be excluded from study participation, receive compensation, and may be referred to an appropriate medical provider. If no spinal segmental dysfunctions are found in one or 2 spinal regions but there is at least one spinal region with a segmental dysfunction present, the participant will be allowed to proceed with the study, and only the spinal segments found will be treated accordingly.

To determine the presence or absence of a spinal segmental dysfunction, MS will assess each spinal region in the following ways:

- Cervical spine (C1): the patient will be seated at the end of the table with their arms relaxed at their sides. The examiner will use their left hand to hold the patient’s head to direct their motion, while their right hand will be performing the following motions: rotation and extension (the examiner contacts the posterior aspect of the C1 transverse process with their right hand and moves the patient through extension and right and left rotation, only once on each side). Coupled motion of C1 is to be used instead of flexion and extension, since the lack of a C1 spinous process makes a flexion and extension assessment of C1 movement challenging. Restriction is indicated at the end of the coupled rotation and extension on the side where there is a “blocked” end feel to the movement (the joint does not glide as far as on the other side).
- Cervical spine (C2-C7): the patient will be seated at the end of the table with their arms relaxed at their sides. The examiner will use their left hand to hold the patient’s head to direct their motion, while their right hand will be performing the following motions: rotation and extension (the examiner contacts the articular pillars bilaterally with the index finger and thumb of their right hand and moves the patient through left and right rotation and extension, feeling for motion of the spinal level). Spinal levels C2 through C7 will be individually assessed and motioned only once on each side. Restriction is indicated when the patient is in rotation and extension on either the left, right, or both sides and there is a “blocked” end feel to the movement (the joint does not glide as far as at other levels).
- Thoracic and lumbar spine: the patient will be seated at the end of the table with their arms relaxed at their sides. The examiner will be stabilizing their right elbow on the patient’s right shoulder to direct their motion, while their left thumb will be pushing along the right lateral aspect of each spinous process to ensure the following motions: extension, rotation, and lateral flexion (as the examiner pushes against the right lateral aspect of the spinous process, they are using the patient’s right shoulder as a lever to assist in the correct movement of the thoracolumbar spine). The examiner will repeat this for the opposite side (standing on the patient’s left, using the left elbow to direct motion, and using the right thumb to palpate the left lateral spinous processes). Restriction is indicated when the patient is in extension, rotation, and lateral flexion on either the left, right, or both sides and there is a “blocked” end feel to the movement (the joint does not glide as far as at other levels).
- Pelvis (Thompson sacral check): the participant will be lying prone on the table with their arms relaxed to the sides. The examiner will stabilize the base of the participant’s sacrum with the base of their palm and ask the participant to raise one leg straight up from the hip as high as they can without bending the knee. After repeating this procedure for the opposite leg, the sacral listing is determined via the Thompson chiropractic protocol, whereby the leg that raises higher is the side of anterior sacral displacement and has a listing of “SAR” if that is the right leg or “SAL” if that is the left leg.
- Pelvis (prone leg check): the participant will be lying prone on the table with their arms relaxed to the sides. The examiner will assess the leg length of the participant in this position. If there is a shorter leg, a listing of the posterior inferior ilium on that side will be interpreted.
- Tenderness to palpation: as the examiner is motioning the patient, they will simultaneously note if there is any discomfort at the levels where motion restriction is found, as this is further indication of a spinal segmental dysfunction [20].
- Soft tissue markers: in addition to noting any soft tissue changes while motioning the patient, the examiner will instruct the patient to lie prone on a flat chiropractic bench and then palpate for soft tissue markers. These soft tissue markers, such as localized edema or taut muscle fibers, will be assessed in the cervical, thoracic, and lumbar spine by gently palpating lateral to the spinous processes in the paraspinal musculature in each region. The sacroiliac joints will be gently palpated in this position as well. The suboccipital muscles will also be palpated along the occipital ridge bilaterally. These soft tissue changes can also indicate a potential spinal segmental dysfunction [20].

Once the determination of a dysfunctional segment in the cervical, thoracic, and lumbopelvic regions has been made, the clinician will notate said listings in the participant file.

### Randomization

Using simple randomization via a box draw, participants will be randomized to one of two arms: (1) genuine high velocity, low amplitude (HVLA) cervical, thoracic, and/or lumbopelvic SMT, or (2) sham HVLA cervical, thoracic, and/or lumbopelvic SMT.

### Sequence Generation

Prior to participant recruitment, 60 blank, opaque, unidentifiable, sealed envelopes will be prepared with an equal distribution of genuine versus sham chiropractic SMT by a lab staff member with no direct contact with participants.

### Concealment Mechanism

To ensure concealment, the envelope allotments for randomization will be kept in the central office in a box that only the clinicians (MS and AJ) will be able to access.

### Implementation (Enrollment and Assignment)

The clinicians (MS and AJ) are responsible for participant enrollment. Following the initial chiropractic exam and immediately prior to the delivery of the participant’s first sham or real SMT, the participant will be permitted to choose their own envelope from a box, though MS or AJ will be the one to open the envelope to review the group assignment. This assignment will be recorded by MS or AJ in a binder that will only be accessible to her throughout the course of the trial.

### Who Will Be Blinded?

Participants, outcome assessors, and data analysts will be unaware of group assignments. The clinicians (MS and AJ) will not be blinded. To improve participant blinding, we will endeavor to make all aspects of the sham sessions identical to the genuine sessions, including the audible drop of the chiropractic table in the cervical, thoracic, and lumbopelvic regions.

### Unblinding Procedure

Group assignments will be disclosed to trial participants 30 days after the completion of the study.

### Assessments

Participants will be asked to attend 2 separate sessions approximately one week apart. Automated text reminders will also be sent via ChiroHD software (ChiroHD Inc) ~24 hours and~3 hours prior to each session.

To assess gait, participants will be asked to walk on a motorized treadmill at their preferred walking speed (PWS). Participants will be fitted with 16 inertial measurement units at different anatomical landmarks. Inertial measurement units will then be calibrated using commercial motion-capture equipment (NORAXON MyoMotion, MR2; Noraxon Inc). Participants will be fitted with a pair of slip-resistant socks prior to testing. They will then be instructed to step onto the nonmoving treadmill to begin the PWS assessment. Participants will start by walking on the treadmill at a speed of 0.5 mph. A trained research investigator will then increase the speed by 0.1 mph increments every 2‐4 seconds until the participant reports that they are walking at their PWS. Then, in 0.1 mph increments, 1 mph is added to the speed, followed by a decrease at a rate of 0.1 mph every 2‐4 sec until they re-establish their PWS. This procedure will be repeated 3 times in total. The speeds will be averaged to determine their PWS. The PWS assessment will only be performed at baseline. Participants will also be blinded to the digital speed display while walking on the treadmill. Once the PWS has been established, participants will be asked to walk on the treadmill for 5 minutes at their PWS. The treadmill will be used to measure forces during walking, as well as several traditional gait parameters (eg, step time, stride time, and steps per minute in milliseconds). Gait assessment during the follow-up session will be identical, except for determining PWS.

### Interventions

The clinicians (MS and AJ) are licensed chiropractors with >20 years of combined experience in providing HVLA treatments. MS was also involved in the creation of the sham protocol. Both clinicians undertook extensive training in its delivery over a ~3 month period.

#### Genuine Chiropractic Treatment

**Figure 2.**
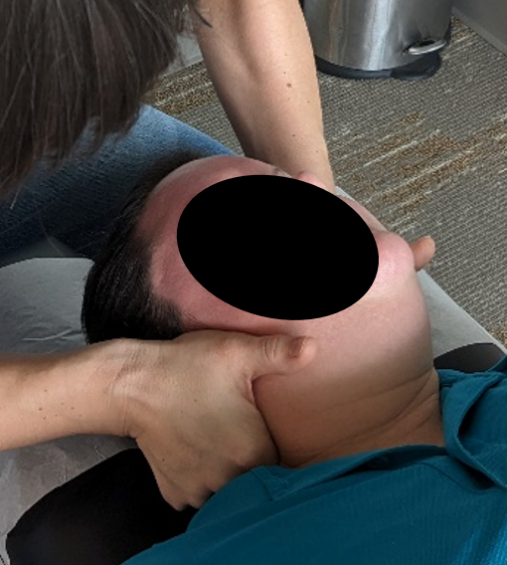
Genuine cervical spinal manipulative therapy (supine cervical set).

**Figure 3.**
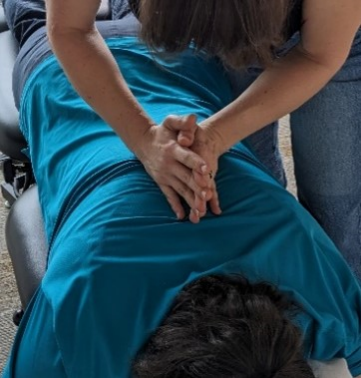
Genuine thoracic spinal manipulative therapy (double hypothenar with drop).

**Figure 4.**
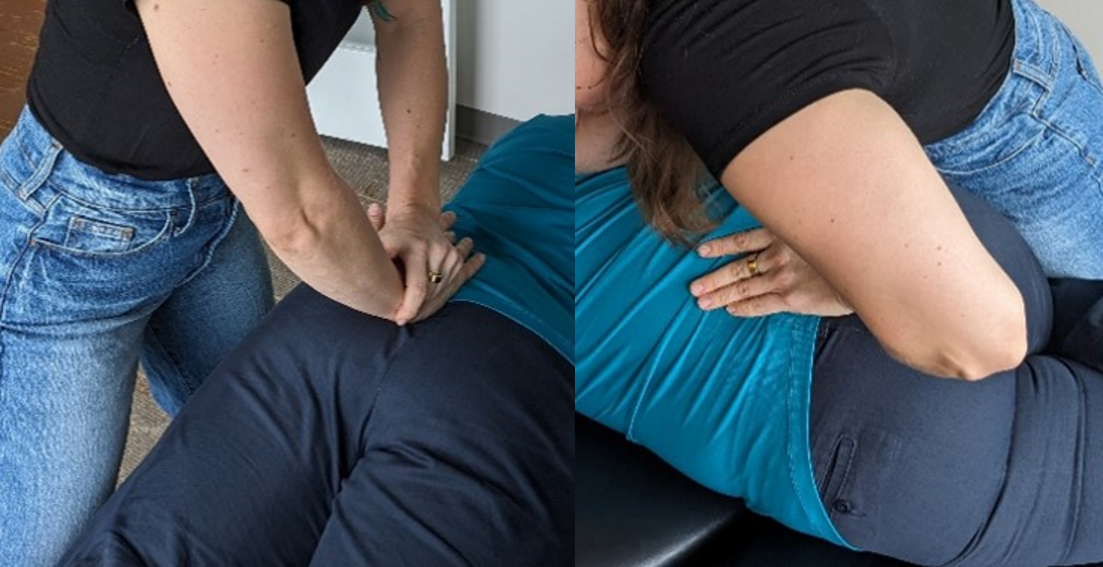
Genuine lumbopelvic spinal manipulative therapy (prone drop or side posture push).

The genuine cervical intervention will be performed in the predetermined location using a supine position and a diversified maneuver (see Figure 2). The thoracic spine will be treated in a prone position using a double hypothenar thrust maneuver (see Figure 3). This will be done with or without an additional drop in the thoracic region at the discretion of the chiropractor and is based on muscle tone in the region. The lumbopelvic region will receive either a prone drop maneuver with 3‐4 drops or a side posture push, depending on clinical findings (see Figure 4). The spinal levels and listings to be treated will be based on examination findings. Only one SMT will be performed in each of the 3 regions assessed (cervical, thoracic, and lumbopelvic). Following the genuine chiropractic treatment, the spine will be reassessed for intersegmental motion restrictions in a postcheck palpation of the spine of the participant. It will be noted in the session notes if the pre- and posttreatment objective findings are the same or different. The levels to be treated may be different at each of the 2 sessions, as determination is based on the palpatory findings at each visit.

#### Sham Chiropractic Treatment

**Figure 5.**
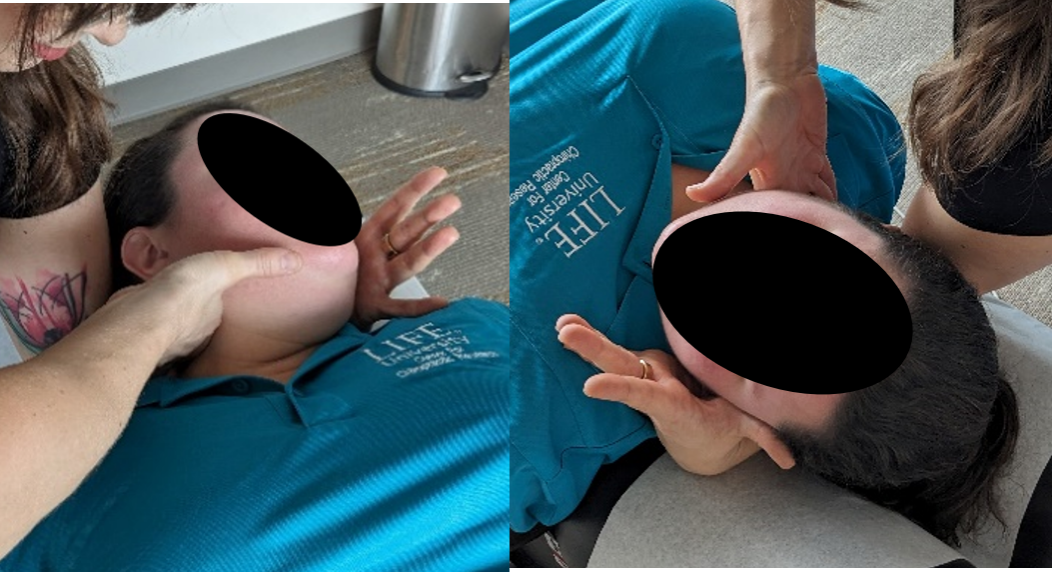
Sham cervical spinal manipulative therapy (supine drop with forearm assist from 2 angles).

**Figure 6.**
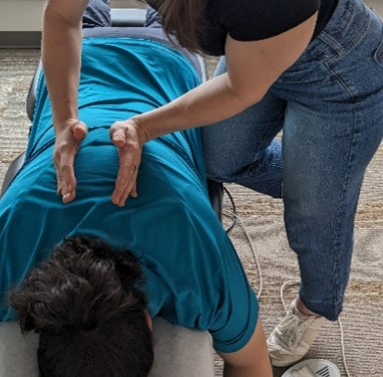
Sham thoracic spinal manipulative therapy (prone drop with knee assist).

**Figure 7.**
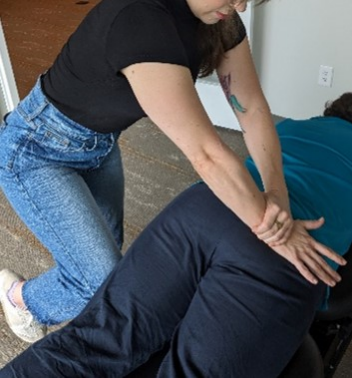
Sham lumbopelvic spinal manipulative therapy (prone drop with knee assist).

For the sham cervical SMT, the participant will be placed in a supine position on a cervical drop piece with the chiropractor’s forearm under the participant’s neck and contact lateral to the cervical spine (see Figure 5). This will only be performed unilaterally over the side with the predetermined restriction in the articular pillars. The thoracic sham (see Figure 6) and the lumbopelvic sham (see Figure 7) will be performed with the participant in the prone position. Contact for the thoracic sham will be medial to the medial scapular border, and contact for the lumbopelvic sham will be over the lateral gluteal muscles, opposite to the side of the chiropractor’s stance. The chiropractor will contact close to the predetermined site of intersegmental motion restriction in the spine, but no manual thrust will be directed into the spine. Instead, the chiropractor will apply a downward pressure to the drop piece with their forearm (cervical) or knee (thoracic and lumbopelvic), which will elicit a noise from the table that mimics a genuine drop SMT, as well as induced movement of the participant’s body. Only a single drop will be made in all regions to limit any potential joint motion with the procedure. Following the sham chiropractic treatment, the spine will be reassessed for intersegmental motion restrictions and objective findings in a postcheck, allowing for the comparison with pretreatment findings. Similar to the genuine treatment, the sham will be performed at levels determined at that particular session.

Table 1 provides a detailed description of the trial interventions using the Template for Intervention Description and Replication (TIDieR) [21]. Immediately following each session, as well as at the beginning of the second session, blinding will be assessed via a 4-item electronic survey taken with the computer program Jotform. Additionally, within 30 days of the trial’s completion, participants will be informed of their group allocation via email.

**Table 1.** Template for intervention description and replication (TIDieR).

Item	Description
Brief name: provide the name or a phrase that describes the intervention.	Drop table-assisted genuine or sham spinal manipulative therapies (SMTs)
Why: describe any rationale, theory, or goal of the elements essential to the intervention.	Using shams in manual therapy (MT) studies allows for comparison of neurological measures (ie, gait parameters) to expose any definitive effects of therapeutic treatments. Blinding efficacy is fundamental to maintaining the validity of said shams. To date, there are limited manual high velocity, low amplitude (HVLA)–mimicking shams that have been validated in MT research.
What (materials): Describe any physical or informational materials used in the intervention, including those provided to participants or used in intervention delivery or training of intervention providers. Provide information on where the materials can be accessed (eg, online appendix or URL).	—^a^
What (procedures): describe each of the procedures, activities, and/or processes used in the intervention, including any enabling or support activities.	Genuine treatment: only one SMT will be performed in each of the 3 regions assessed (cervical, thoracic, and lumbopelvic). The genuine cervical intervention will be performed in the predetermined location using a supine position and a diversified maneuver. The thoracic spine will be treated in a prone position using a double hypothenar thrust maneuver. This will be done with or without an additional drop in the thoracic region at the discretion of the chiropractor and was based on muscle tone in the region. The lumbopelvic region will receive either a prone drop maneuver with 3-4 drops or a side posture push, depending on clinical findings. Sham treatment: only one sham will be performed in each of the 3 regions assessed (cervical, thoracic, and lumbopelvic). For the sham cervical SMT, the participant will be placed in a supine position on a cervical drop piece with the chiropractor’s forearm under the participant’s neck and contact lateral to the cervical spine. This will only be performed unilaterally over the side with the predetermined restriction in the articular pillars. The thoracic sham and the lumbopelvic sham will be performed with the participant in the prone position. Contact for the thoracic sham will be medial to the medial scapular border, and contact for the lumbopelvic sham will be over the lateral gluteal muscles, opposite to the side of the chiropractor’s stance. The chiropractor will contact close to the predetermined site of intersegmental motion restriction in the spine, but no manual thrust will be directed into the spine. Instead, the chiropractor will apply a downward pressure to the drop piece with their forearm (cervical) or knee (thoracic and lumbopelvic), which will elicit a noise from the table that mimics a genuine drop SMT, as well as induced movement of the participant’s body. Only a single drop will be made in all regions to limit any potential joint motion with the procedure.
Who Provided: for each category of intervention provider (eg, psychologist and nursing assistant), describe their expertise, background, and any specific training given.	Georgia-licensed Doctors of Chiropractic (DCs) with 20+ years of combined experience in the delivery of HVLA SMT and ~3 months of sham protocol training.
How: describe the modes of delivery (eg, face-to-face or by some other mechanism, such as internet or telephone) of the intervention and whether it was provided individually or in a group.	Genuine and sham SMTs will be performed individually and face-to-face.
Where: describe the type(s) of location(s) where the intervention occurred, including any necessary infrastructure or relevant features.	Sessions will take place at the Dr Sid E. Williams Center for Chiropractic Research (CCR), Life University, Marietta, Georgia, United States.
When and how much: describe the number of times the intervention was delivered and over what period including the number of sessions, their schedule, and their duration, intensity, or dose.	Over the course of a ~1-week period, participants will attend 2 sessions (first ~2 hours, second ~1 hour) scheduled at approximately the same time of day.
Tailoring: if the intervention was planned to be personalized, titrated, or adapted, then describe what, why, when, and how.	Genuine and sham SMTs will be delivered to areas of spinal segmental dysfunction as determined by the chiropractic clinician using an established, reliable detection protocol.
Modifications: if the intervention was modified during the study, describe the changes (what, why, when, and how).	—
How well (planned): if intervention adherence or fidelity was assessed, describe how and by whom, and if any strategies were used to maintain or improve fidelity, describe them.	Participant adherence to the intervention schedule will be monitored by the chiropractic clinician. Attempts will be made to mitigate adherence issues via automated SMS text messaging reminders sent ~24 and ~3 hours prior to each session.
How well (actual): if intervention adherence or fidelity was assessed, describe the extent to which the intervention was delivered as planned.	—

a Not applicable.

### Criteria for Discontinuing or Modifying Allocated Interventions

Participants will be advised that they are able to withdraw at any time without giving a reason or may be withdrawn by the investigators if they experience significant adverse effects that are deemed detrimental to their well-being, as determined by the clinician.

### Strategies to Improve Adherence to Interventions

We will attempt to mitigate adherence issues via automated SMS text messaging reminders sent ~24 hours and ~3 hours prior to each session.

### Plans to Promote Participant Retention and Complete Follow-Up

Once enrolled, every reasonable effort will be made to follow participants throughout the entirety of the study period via ongoing email and SMS text messaging correspondence. In the event of premature discontinuation of the study for any reason, participants will be made aware that all data collected up to the point of withdrawal may be used for analyses, though they will have the right to pull their data if so desired.

### Relevant Concomitant Care Permitted or Prohibited During the Trial

Participants will be asked to avoid any outside manual therapies (eg, chiropractic and osteopathy) from 14 days prior to their initial session until the end of the trial period. Any introduction of manual therapies during this period will render participants ineligible.

### Measurements (All Assessments Will Be Performed by the Data Team)

#### Primary Outcome

Blinding will be assessed immediately following each session and directly prior to the second session via a brief electronic survey whereby participants will be asked (1) their perceived group allocation (“genuine adjustments,” “sham adjustments,” and “I don’t know”), (2) if their group assignment was revealed to them in any way (“No,” “Yes - explain”), (3) if they felt any changes in their body following their sessions (“No,” “Yes-explain,” and “I don’t know”), and (4) if they have received chiropractic care prior to this study (“No” and “Yes-for how long-what kind”). This data will be stored in Jotform. The Bang BI is a continuous value from −1 to 1 computed on each trial arm [22]. Per the index developers, an index score of 1 indicates that all responses are correct, suggesting complete unblinding. An index of −1 denotes that all responses are incorrect, suggesting either complete blinding or unblinding in the opposite direction if participants have some bias leading them to believe they are in the opposite arm. An index of 0 indicates half of the responses are correct and half are incorrect, which suggests random guessing. Arm-level Bang BIs between −0.3 and 0.3 are commonly interpreted as “successful blinding” [14-16]. Successful blinding might also be inferred in cases of “wishful thinking,” whereby trial participants tend to believe they received genuine treatment irrespective of group assignment, resulting in Bang BIs for the genuine and sham groups approaching 1 and −1, respectively (summed Bang BI ≈0) [23].

#### Secondary Outcomes

##### James BI

The James BI will also be used for evaluating the blinding success of the participants based on their electronic survey answers as noted above. The James BI is calculated based on the proportion of participants who correctly or incorrectly guess their treatment group, where a “don’t know” response is considered a successful blinding outcome [22]. This index is scored on a scale of 0-1; a score of 0 indicates complete lack of blinding, while a value of 1 indicates perfect blinding. Generally, acceptable blinding is indicated when values are between 0.5 and 1 [13].

##### Gait

Secondary outcomes will assess the effects of spinal manipulative therapy on spatiotemporal gait characteristics. Gait will be evaluated using inertial measurement motion capture units and an instrumented force-plate treadmill (Noraxon USA Inc). Spatiotemporal gait parameters will include step length, step time, cadence, and velocity. Gait events will be identified using vertical ground reaction force thresholds implemented within Noraxon’s analytical pipeline. Where applicable, parameters will be processed bilaterally and averaged to generate representative values for each participant. Gait will be evaluated at baseline and at the end of the second session to assess changes over time, as well as differences between participants receiving genuine SMT versus sham SMT.

### Sample Size

As recommended in the literature [24], a precision-based approach (ie, 95% CI width) was used to determine the sample size. Using a custom R script (R Foundation for Statistical Computing) developed by Muñoz Laguna et al [15] for a similar study [16], it was estimated that a sample size of ~25 participants per arm would yield Bang BI 95% CI widths of 0.45. Allowing for ~15% loss to follow-up, we computed an adjusted sample size of ~30 participants per arm (ie, 25/[1.0‐0.15]=~30).

### Statistical Considerations (Primary Outcome)

The primary focus will be on arm-specific blinding, which will be reported using Bang BI point estimates and 95% CIs computed using a custom script written in “R” (v4.4.3) [25], which incorporates the “BI” package [26].

### Statistical Considerations (Secondary Outcomes)

As our sample size determination was based on our primary outcome (ie, Bang BI), estimates derived from secondary end points will be considered exploratory and interpreted with caution. Additionally, to guard against over-interpretation of results, we will only perform estimation and not perform null hypothesis significance testing. James BI point estimates and 95% CIs will be constructed using a custom R script incorporating the “BI” package. For group-level analysis of other secondary outcomes, mixed models will be run via custom R scripts incorporating the lme4 package [27]. Mixed models account for nonindependent data (eg, repeated measures) and have been shown to provide robust estimates in longitudinal designs even with high levels of missing data [28]. Between-group change score differences over time will be evaluated using linear mixed models with random intercepts adjusting for age, sex, and baseline values.


$$\text{lmm}=\text{lmer}\;\left(y-y_{\text{baseline}}∼1+\text{time}*\text{group}+y_{\text{baseline}}+\text{age}+\text{sex}+(1|\text{ID}),\;\text{data}=d\right)$$

Assumption checks will be performed via “visualize()” in the flexplot package [29,30]. If model assumptions are severely violated, data were evaluated via generalized linear mixed models incorporating a gamma distribution and a log link.


$$\text{glmm}=\text{glmer}\;\left(y-y_{\text{baseline}}∼1+\text{time}*\text{group}+y_{\text{baseline}}+\text{age}+\text{sex}+(1|\text{ID}),\;\text{data}=d,\;\text{family}=\Gamma (\text{link}=\log)\right)$$

Between-group change score difference point estimates and 95% CIs will be computed and plotted using the emmeans package [31]. Gait parameters include multiple correlated measures. Therefore, Bonferroni correction will be applied to account for multiplicity and control the family-wise type I error rate.

### Methods in Analysis to Handle Protocol Nonadherence and Any Statistical Methods to Handle Missing Data

We will report the number and percentages of withdrawals in each of the groups. Based on the trialists’ experience, loss to follow-up is expected to be no more than 15%, which has been factored into our sample size calculation. Regarding secondary outcomes, mixed models have been shown to provide robust estimates in longitudinal designs even with high levels of missing data.

### Adverse Event Reporting and Harms

The principal investigator (PI) will be responsible for ensuring participants’ safety and for reporting anticipated adverse events, unanticipated adverse events (UAEs), unanticipated problems (UPs), and serious adverse events (SAEs) to the institutional review board (IRB). Once the PI is made aware of any SAEs, UAEs, or UPs by study personnel, she will prepare and send a report to the IRB within 5 business days. Anticipated adverse events will be reported in the annual continuing review application. In response to adverse events (AEs) and UPs, the PI may choose to incorporate addendums into the study protocol to mitigate risks. If the PI determines that there is imminent danger to the trial participants, she may terminate the study only following consultation with other trialists. If this occurs, the IRB and participants will be immediately notified, and all data collected up to the point of suspension or termination may be used in the final analyses. A list of any reported AEs, UAEs, UPs, and SAEs will be logged and maintained by a lab team member.

### Data Management and Processing

To ensure data integrity, trialists review protocols monthly to ensure the procedures for storing and sharing data are being followed. The results from the trial will be reported in aggregate, and the data will be retained for a minimum of 10 years.

### Access to Data

The final trial dataset will be password protected and housed locally at the CCR. Oversight officials (eg, Life University’s IRB) may also be given access to the files upon request.

### Plans to Give Access to the Full Protocol, Participant-Level Data, and Statistical Code

The full trial protocol will be submitted for publication to a peer-reviewed, open-access journal prior to the completion of data collection and analyses. In line with scientific imperatives of increased transparency, reproducibility, and interpretations of trials, statistical codes and deidentified data sets will be provided to interested researchers upon request.

### Oversight and Monitoring

#### Composition of the Data Monitoring Committee, Its Role, and Reporting Structure

No independent data and safety monitoring board was required for this trial. Per the CCR’s standard policies developed to ensure good trial practices and identify emerging trends, our lab has in place a data and safety monitoring plan whereby trialists review the data collection procedures, AEs, and trial progress once monthly.

#### Frequency and Plans for Auditing Trial Conduct

Auditing of trial conduct will be performed once monthly by the trialists.

#### Plans for Communicating Important Protocol Amendments to Relevant Parties (eg, Trial Participants and Ethical Committees)

Substantive protocol amendments that may impact the conduct of the study will be agreed upon by the research team, submitted to the IRB for approval, reported in the trial registry, reflected in the participant-facing materials (eg, informed consent documents), and outlined in the final manuscript.

#### Dissemination Policy

Every effort will be made to minimize the interval between the completion of data collection and the release of study results. We estimate this process to take up to 12 months. Irrespective of the magnitude or direction of effect, results from the study will be submitted for publication in an international peer-reviewed scientific journal, presented at scientific conferences, and may form part of future grant applications.

### Ethical Considerations

Ethical approval was obtained from the IRB of Life University (2025-03-27-Sliwka; approved on March 27, 2025; 1269 Barclay Cir, Marietta, GA 30060). This is a protocol, and no participants have been recruited to date for the sake of this manuscript. Participants will receive up to US $75 in gift cards to compensate for their time (a US $25 gift card after the first session and a US $50 gift card after the second session). Photo releases for images in this manuscript have been procured.

Written informed consent will be collected at Life University’s CCR. At the initial meeting, a research assistant will (1) provide each potential participant with a paper copy of the informed consent form written in English, (2) outline the key aspects of the trial, (3) ask if they have any questions about the trial, and (4) request the participant’s signature. Participants will be informed that they may withdraw at any time without giving a reason and that all data collected up to the point of withdrawal may be used in the final analysis.

All information generated in this study will be considered highly confidential and is not to be shared with any persons not directly concerned with the trial. For deidentification purposes, participants will be assigned unique study numbers upon enrollment. Apart from the health history forms, all electronic records will be identified solely using assigned study numbers and stored locally in a password-protected database. Any records that contain personal identifiers (ie, informed consent forms, health history forms) will be stored on-site in a locked office or locally in a password-protected database accessible only to the researchers directly involved in the trial.

## Results

This study is being supported and internally funded by the Dr. Sid E. Williams Center for Chiropractic Research at Life University in Marietta, Georgia, United States. Our study was prospectively registered on clinicaltrials.gov (NCT06931600) on March 28, 2025, and the first participant was seen on May 5, 2025. As of February 11, 2026, 25 participants have been enrolled. Projections for completing data collection, data analysis, and manuscript submission are Summer 2026, Fall 2026, and Winter 2027, respectively.

## Discussion

### Overview

Though health care clinical trials tend to rely on RCTs for treatment analysis, creating an inert yet believable sham for SMT trials has proven challenging [1,3]. Despite attempts using techniques ranging from light touch to simulated thrusts, there is no universally recognized manual sham for SMT trials, and few sham techniques account for participant blinding [6-12].

### Principal Findings

To address the lack of a standardized manual SMT sham, our lab will launch a study testing a novel full-spine manual SMT sham. Our main goal is to assess the blinding success of this novel sham. Secondary aims are to explore the possible effect(s) of SMT on gait parameters by comparing movement characteristics and gait-related motor control between individuals receiving genuine SMT and those receiving sham SMT.

### Comparison to Prior Work

This multisession trial is the second step in our lab’s quest to develop a standardized sham protocol. Previously, a feasibility study was conducted in our lab that assessed blinding success following a single session of the same sham and genuine chiropractic techniques mentioned in this protocol. Bang BI point estimates suggested a high level of unblinding in the genuine group but successful blinding in the sham, both immediately posttreatment as well as 48 hours later. We hypothesize similar results from this study.

### Strengths and Limitations

One potential limitation of our protocol is that we are not undertaking a crossover design. By limiting participants to one study arm, they will not receive both genuine and sham SMT, therefore not controlling for between-subject variability. However, since manual therapies may have long-lasting neurophysiological effects [32], a parallel study can avoid carryover effects. When it comes to gait, there is a high variability due to individual differences, which we will account for using a generalized linear mixed model. Another potential limitation relates to our recruitment of participants from a geographic area with a population that is more familiar with chiropractic care due to the local chiropractic college. We will exclude participants with substantial chiropractic education to help mitigate this potential bias.

### Future Directions

Ultimately, this line of research is endeavoring to develop a novel manual SMT sham protocol that can be used to improve the quality of future chiropractic RCTs that use clinical measures as primary outcomes.

### Dissemination Plan

Regardless of outcomes, the findings will be published in an international peer-reviewed journal within 12 months of trial completion.

## Abbreviations

Bang BI: Bang Blinding Index
CCR: Center for Chiropractic Research
HVLA: high velocity, low amplitude
IRB: institutional review board
James BI: James Blinding Index
PI: principal investigator
PWS: preferred walking speed
RCT: randomized controlled trial
SAE: serious adverse event
SMT: spinal manipulative therapy
TIDieR: Template for Intervention Description and Replication
UAE: unanticipated adverse event
UP: unanticipated problem
